# A Comparison of Skin Staining after Sentinel Lymph Node Biopsy in Women Undergoing Breast Cancer Surgery Using Blue Dye and Superparamagnetic Iron Oxide Nanoparticle (SPIO) Tracers

**DOI:** 10.3390/cancers14236017

**Published:** 2022-12-06

**Authors:** Allan Jazrawi, Madeleine Wärnberg, Abdi-Fatah Hersi, Christine Obondo, Lida Pistioli, Staffan Eriksson, Andreas Karakatsanis, Fredrik Wärnberg

**Affiliations:** 1 Centre for Clinical Research, County Västmanland, Uppsala University, 721 89 Västerås, Sweden; 2 Department of Surgery, Västmanlands County Hospital, 721 89 Västerås, Sweden; 3 Department of Surgical Sciences, Uppsala University, 751 85 Uppsala, Sweden; 4 Department of Plastic Surgery, Institute of Clinical Sciences, Sahlgrenska Academy, University of Gothenburg, 413 45 Gothenburg, Sweden; 5 Department of Surgery, Södersjukhuset, 118 83 Stockholm, Sweden; 6 Department of Surgery, Institute of Clinical Sciences, Sahlgrenska Academy, University of Gothenburg, 413 45 Gothenburg, Sweden; 7 Department of Surgery, Section for Endocrine and Breast Surgery, Uppsala University Hospital, Akademiska Sjukhuset, 751 85 Uppsala, Sweden

**Keywords:** sentinel lymph node biopsy, breast cancer, blue dye, superparamagnetic iron oxide, magnetic tracer, sentinel lymph node, skin staining

## Abstract

**Simple Summary:**

Both superparamagnetic iron oxide nanoparticles (SPIO) and blue dye (BD) have been reported to cause skin staining after breast-conserving surgery. SPIO is a novel tracer that has been shown to identify sentinel lymph nodes (SLNs) in patients with breast cancer. Our study was the first to compare the incidence and size of skin staining between the two tracers. We reported on these outcomes in a preplanned secondary analysis of a prospective clinical trial in which women received both SPIO and BD. This study investigated whether there was a difference in the incidence and size of skin staining between SPIO and BD after SLN-dissection. In all, 270 women were operated on with breast-conserving surgery and received SPIO, and 204 of these women also received BD. After 24 months of follow up, there was no statistically significant difference between the two tracers with regard to the size and incidence of skin staining.

**Abstract:**

Superparamagnetic iron oxide nanoparticles (SPIO) are a tracer for sentinel lymph node (SLN) detection. In a preplanned secondary analysis of a prospective clinical trial (SentiDose) we reported on skin staining after SPIO and blue dye (BD) injections. For SPIO, either a 1.5 mL retroareolar injection on the day of surgery or a 1.0 mL peritumoral/retroareolar injection 1–7 days before surgery was given. A 1.0 mL sub-/intradermal periareolar injection of BD was also administered to all these women. Staining was then assessed at 6, 12 and 24 months after surgery. A total of 270 women received SPIO and were operated on with breast-conserving surgery. Of these, 204 women also received BD. A total of 58 (21.5%) women had an SPIO stain 6 months postoperatively with a median size of 6.8 cm^2^ (*p* = 0.56), while 51 (25.0%) had a BD stain with a median size of 8.5 cm^2^ (*p* = 0.93). The incidence and size of SPIO and BD staining decreased over time reciprocally. At 24 months, the incidence and median size of SPIO was 23 (8.6%) and 4 cm^2^, respectively. For BD, the incidence was 14 (6.3%, *p* = 0.13), and the median size was 3.5 cm^2^ (*p* = 0.18). There was, therefore, no statistically significant difference in the incidence or size of skin staining between SPIO and BD over time.

## 1. Introduction

Sentinel lymph node dissection (SLND) constitutes the standard of care for axillary staging in patients with clinically node-negative early breast cancer, as it is accurate and associated with a decreased morbidity compared to the historical standard of axillary lymph node dissection (ALND) [1,2,3,4,5]. Novel tracers have been developed to overcome the limitations of radioactive isotope technetium99 (Tc^99^)- and Patent Blue V^®^ (BD)-based detection, such as a short Tc^99^ half-life, strict regulations in handling and disposal, access to medical facilities and allergic reactions [6,7,8,9]. SPIO is a tracer with a comparable performance in previous studies and meta-analyses [6,7,8,9]. It is logistically convenient to use, as the timing of its administration is flexible [10,11,12]. 

Skin staining after an SPIO injection has previously been a concern as has been the case with BD [10,11,13,14]. However, the bulk of studies reporting on SPIO investigated this outcome after a retroareolar superficial injection of 2.0 mL of SPIO diluted in 3 mL of NaCl [10,11]. Since then, other studies have reported on smaller doses of SPIO (undiluted 2.0, 1.5 and 1.0 mL) in different time frames and different injection sites (peritumoral) [15]. In the SentiDose multicenter trial [15], patients received either 1.5 mL in the subareolar area less than 24 h before surgery or 1.0 mL 1–7 days before surgery. The injection site was left to the discretion of the operating surgeon. All patients however received Tc^99^ +/− BD as a background control. In this study, the SLN detection rates at lower doses were found to be comparable to Tc^99^ +/− BD. Following a 1.5 mL retroareolar injection, the detection rate was 97.5%, and it was 100% after a 1.0 mL peritumoral injection. This was comparable to Tc^99^ +/− BD. The longitudinal follow-up of skin staining after breast-conserving surgery with SPIO and BD were predefined secondary endpoints. 

The primary outcome of this predefined analysis from the SentiDose study (SentiColor subprotocol) was to report on the incidence, duration and size of skin staining in the SentiDose patient population injected with both SPIO and BD. The secondary outcomes were to determine the predictive factors for SPIO staining and to investigate if different injection techniques could prevent skin staining.

## 2. Materials and Methods

### 2.1. Patient Selection

Between 2017 and 2019, all women in the SentiDose trial [15] undergoing breast-conserving surgery (BCS) and SLNB at six Swedish Hospitals were recruited into this study. Both SPIO and Tc^99^ were used in all women, while BD was used as an adjunct according to local routines or surgeon preferences. Inclusion criteria were primary breast cancer (cT0–2cN0cM0) and Eastern Cooperative Oncology Group (ECOG) performance status 0–2. Women with previous ipsilateral breast or axillary surgery and/or radiation and neoadjuvant chemotherapy were excluded. Pregnant women, patients with iron sickness, patients who underwent mastectomy either primarily or within six months of initial surgery were excluded in this follow-up part of the SentiDose trial. The study was approved by the Uppsala University regional ethics committee (Decision Number 2017/063).

### 2.2. Methods

#### 2.2.1. Procedure

In the SentiDose trial, SLN detection rates were compared after injecting two different doses of SiennaXP^®^/Magtrace^®^ (Sysmex Europe, Hamburg, Germany) at different time points using different injection techniques [15]. SPIO was injected either as a 1.5 mL retroareolar injection at least 20 min preoperatively on the day of surgery or as a 1.0 mL peritumoral/retroareolar injection one to seven days before surgery. The 1.5 mL dose was followed by a five-minute massage, whereas the massage was optional for the 1.0 mL dose. As a control measure, all the women also received Tc^99^ +/− BD according to each site’s routine. BD was given as a 1.0 mL sub-/intradermal or retroareolar injection.

The Sentimag probe was used during surgery to localize the SLN, and a gamma probe was subsequently used to identify any residual SLNs with radioactive signal. All magnetic, radioactive, blue, or brown SLNs were excised. The conventional cutoff of 10% of the SLNs with the highest signal (SPIO or Tc^99^) was implemented to define additional SLNs. 

#### 2.2.2. Data Collection

Patient and tumor characteristics are presented in Table 1. The incidence of skin staining and the size of the staining in square centimeters (cm^2^) were followed up by telephone interviews 6, 12 and 24 months after surgery. If there was no staining, brown (SPIO) or blue (BD), on the first visit three to four weeks after surgery, no further follow-up was conducted. Follow-up was also ended when staining disappeared or at 24 months. Mean staining size calculations included only women with a stain at each time point. SLN detection rates have been reported elsewhere [15]. This manuscript was prepared according to the Strengthening the Reporting of Observational Studies (STROBE) Statement [16].

#### 2.2.3. Statistical Analysis

Staining was analyzed in women with BCS. Descriptive statistics were performed with means (95% confidence interval) or medians (range) of continuous variables, and, depending on data distribution, statistical analyses were based on medians. Continuous data were analyzed using nonparametric tests. Dichotomous data were analyzed with Pearson chi-square for nonpaired observations and McNemar’s test for paired observations. Spearman´s rho test was used to measure the correlation between predictive factors for skin staining. Data analyses were performed using SPSS^®^ (V 26.0. Armonk, NY, USA, IBM Corp.). 

## 3. Results

In total, 271 women were operated on with BCS in the SentiDose trial, and one woman was later excluded due to a conversion to a mastectomy. Patient characteristics are presented in Table 1. SPIO was given to 270 patients. A total of 129 of these had a 1.5 mL retroareolar injection on the day of surgery, 71 patients had a 1.0 mL retroareolar injection 1–7 days before surgery, and 70 patients had a 1.0 mL peritumoral injection 1–7 days before surgery. In addition, 204 of the 270 women also received BD (76%) (95% confidence interval 0.70–0.81). 

At six months, 58/270 (21.5%) women had an SPIO skin stain with a mean size of 12.6 cm^2^ (95% confidence interval 5.8–19.5) and a median of 6.8 cm^2^ (range of 1–88). Between the 6- and 12-month controls, one woman had a mastectomy, and one died. Both of these women had an SPIO stain but no BD stain at 6 months. The corresponding data for SPIO staining at 12 and 24 months were 41/268 (15.3%) with a mean size of 5.9 cm^2^ (95% confidence interval 1.3–10.4) and a median of 4 cm^2^ (range of 1–28 cm^2^) and 23/268 (8.6%) with a mean size of 6.4 cm^2^ (95% confidence interval 0.7–12.0) and a median of 4 cm^2^ (range of 0–20 cm^2^) (Table 2). At six months, 51/204 (25%) women had a BD skin stain with a mean size of 10.8 cm^2^ (95% confidence interval 4.4–17.1) and a median of 8.5 cm^2^ (range of 3–25 cm^2^). Between the 6- and 12-month controls, one woman with a BD stain but without an SPIO stain was lost to follow-up. The corresponding data for BD staining at 12 and 24 months were 32/201 (15.9%) with a mean size of 4.2 cm^2^ (95% confidence interval 1.4–7.1) and a median of 4 cm^2^ (range of 0–9 cm^2^) and 13/201 (6.3%) with a mean size of 4.9 cm^2^ (95% confidence interval 0.4–9.4) and a median of 3.5 cm^2^ (0–15 cm^2^) (Table 2). When comparing the incidence and size of the skin staining with SPIO and BD after 6, 12 and 24 months, there was no statistically significant difference between the two tracers (Table 2). There was a significant reduction in the incidence of SPIO-induced skin staining between 6 vs. 12 months (21.5% vs. 15.3%; *p*-value of >0.0005) and 12 vs. 24 months (15.3% vs. 8.6%; *p*-value of >0.0005). The trend was similar for BD with the following corresponding numbers 25% vs. 15.9% and 15.9% vs. 6.3% with a respective *p*-value of >0.0005 for both comparisons. 

With regard to SPIO dosing, there was a significant difference between the 1.5 mL and 1.0 mL SPIO cohorts with reference to the incidence of skin staining at 6 months; there was an incidence of 34/129 (26.4%) versus 24/141 (17%) (*p* = 0.011) (Table 3). The corresponding numbers at 12 and 24 months were 23/128 (18%) and 13/128 (10.2%) versus 18/140 (12.9%) and 10/140 (7.1%) (*p* = 0.05 and *p* = 0.034, respectively). The median size of the skin staining for the separate volumes and injection techniques for SPIO are shown in Table 3. Only a small portion of women had a 1.0 mL peritumoral injection, but, in this subcohort, we noted the lowest incidence and the smallest stains (see Table 3). However, due to the low numbers, we did not look at the statistical differences between the 1.5 mL injection cohort and the 1.0 mL retroareolar injection cohort. 

**Figure 1:**
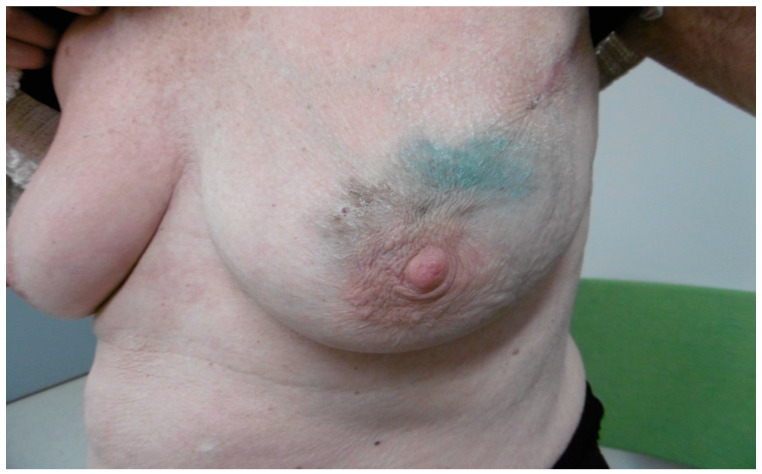
Illustrates a woman 6 months postoperative who received both SPIO and BD. The blue color represents BD staining. The brown color represents SPIO staining.

A low BMI showed a statistically significant positive correlation with skin staining at 6 months of 0.176 (Spearman’s rho) (95% confidence interval 0.054–0.292) (*p* = 0.004), but the difference had disappeared at 24 months to 0.098 (Spearman’s rho) (95% confidence interval 0.026–0.218) (*p* = 0.111). Figure 1 below shows a patient who had both SPIO and BD staining at 6 months.

## 4. Discussion

This study is, to our knowledge, the largest prospective study in which the incidence of skin staining with both SPIO and BD were followed up in parallel. In this study, more than 200 women received both SPIO and BD. There were no significant differences in incidence or size of skin staining when comparing SPIO and BD as tracers for SLNB. After 6 months, the incidence was 21.5% with a median size of 6.8 cm^2^ for SPIO and 25% with a median size of 8.5 cm^2^ for BD. Both SPIO and BD stains diminished in a similar fashion over time. The incidence and median size of SPIO staining at 24 months was 8.6% (4.0 cm^2^) and 6.3% (3.5 cm^2^) for BD.

A lower volume of SPIO resulted in lower rates and smaller sizes of skin staining that disappeared faster. A peritumoral injection resulted in a lower incidence of skin staining compared to a retroareolar injection. The lowest incidence and smallest skin stains were noted when a 1.0 mL peritumoral injection was used. However, no statistical analysis was carried out in this subgroup due to the low number of women in the group. Further, a low preoperative BMI was a predictive factor for an increased risk of skin staining. A possible explanation is that the dispersion of SPIO in a larger breast seems to result in less uptake by the skin lymphatics and the removal of the bulk of SPIO when performing the BCS. 

In earlier studies, 35 to 41% of women were reported to have BD staining at 12 months along with 8.6% after 36 months. However, none of the women reported a cosmetic or psychological problem relating to the BD staining [13,14]. Long-lasting skin staining has previously been reported after injection of SPIO. In the SUNRISE study by Rubio et al., [17] a retroareolar injection of 1.0–1.5–2.0 mL of SiennaXP was used, and 70.3% of the women undergoing a breast-conserving surgery reported discoloration one month after surgery. The staining diminished over time, and the majority of women did not regard the SPIO discoloration as a problem. It has also been shown that by modifying the injection technique, discoloration can be reduced. In an earlier cohort we showed that a deeper peritumoral injection reduced SPIO staining compared to a retroareolar injection: 37.8% and 67.3%, respectively [11].

The preoperative injection of SPIO compared to perioperative administration was associated with the identification of more SNs, and no extra intraoperative time or a massage at the injection site was required for the tracer to migrate to the axilla. This resulted in a shorter operating time with no consequent problems relating to tracer spillage or the diminished visualization of the SN as experienced with other methods such as t ICG [10].

A further concern with SPIO usage is the likelihood of MRI artifacts due to SPIO´s paramagnetic properties [18,19]. A peritumoral injection results in the excision of most of the SPIO used during surgery as discussed by Ghilli et al. [20]. The latter may result in a reduction of MRI artifacts although this remains to be proven. Our research group is currently addressing this issue in the prospective POSTMAG MRI trial [21].

A potential limitation in our study was the possible risk of reporting bias. The follow-up was conducted by telephone interviews. The initial status of staining at the first follow-up at two to four weeks after surgery was documented in the clinical register form (eCRF). If the medical record clearly stated that no stain was visible, then no further follow-up was initiated. Furthermore, the study protocol did not mandate the use of BD. While this restricted the numbers in the study, it was more in line with real world data that show that surgeons familiar with the isotope are often hesitant about adding BD to avoid its known adverse effects. It might have been that the use of BD in all the patients could have affected the reported outcomes, but this was a conscious decision of the investigators to align with optimal patient outcomes.

## 5. Conclusions

To summarize, no differences in either incidence or size of skin staining were noted when comparing SPIO and BD after 6, 12 and 24 months of follow up. For both tracers, the staining diminished or disappeared in a similar manner over time. A lower dose of SPIO resulted in a lower incidence, a smaller size and faster diminishing of staining. With the benefits of no need for nuclear medical facilities, detection rates similar to the dual technique and the possibility to inject well before surgery, SPIO appears to be an appealing tracer choice for sentinel lymph node surgery.

## Figures and Tables

**Table 1:** Patient and tumor characteristics in women undergoing breast-conserving surgery and sentinel lymph node biopsy.

Patient Characteristics	
Patient age at operation (median, range)	64 (38–87)
Body mass index (median, range)	26.3 (16.8–49.3)
Menopausal status, *n* (%)	
Premenopausal	54 (20)
Postmenopausal	215 (79.6)
Missing data	1 (0.4)
Preoperative tumor size, mm median (range)	16 (0–93)
Tumor localization, *n* (%)	
Upper outer	130 (48.1)
Upper inner	51 (18.9)
Lower outer	32 (11.9)
Lower inner	24 (8.9)
Central	33 (12.2)
Histological type, *n* (%)	
Invasive ductal	197 (73)
Invasive lobular	41 (15.2)
Other histology	32 (11.8)
Type of axillary surgery, *n* (%)	
Sentinel lymph node dissection	265 (98.1)
Axillary lymph node dissection	5 (1.9)

**Table 2:** Incidence and median size of postoperative skin staining in women treated with breast-conserving surgery and injected with superparamagnetic nanoparticles of iron oxide (SPIO) and Blue Dye (BD) for sentinel lymph node detection.

Staining	SPIO *n* = 270	BD *n* = 204	*p*-Value
Incidence, number (%)			
6 months	58 (21.5%)	51 (25%)	0.556 ^a^
12 months	41(15.3%)	32 (15.9%)	0.430 ^a^
24 months	23 (8.6%)	13 (6.3%)	0.132 ^a^
Median Size, cm^2^ (range)			
6 months	6.8 cm^2^ (1–88)	8.5 cm^2^ (3–25)	0.925 ^b^
12 months	4 cm^2^ (1–28)	4 cm^2^ (0–9)	0.345 ^b^
24 months	4 cm^2^ (0–20)	3.5 cm^2^ (0–15)	0.176 ^b^

^a^ McNemar test; ^b^ Wilcoxon signed rank test.

**Table 3:** Incidence and median size of SPIO staining postoperatively by injection site and volume.

		Injection Site	
SPIO stain	Retroareolar	Retroareolar	Peritumoral
	1.5 mL, *n* = 129	1.0 mL, *n* = 71	1.0 mL, *n* = 70
6 months	34/129 (26.4%)	16/71 (22.5%)	8/70 (11.4%)
12 months	23/128 (18%)	12/70 (17.1%)	6/70 (8.6%)
24 months	13/128 (10.2%)	8/70 (11.4%)	2/70 (2.9%)
Median Size, cm^2^ (range)			
6 months	8.5 cm^2^ (1–64)	6 cm^2^ (1–88)	8.5 cm^2^ (1–25)
12 months	4 cm^2^ (1–28)	4 cm^2^ (1–28)	6.8 cm^2^ (3–9)
24 months	5 cm^2^ (0–20)	4 cm^2^ (0–20)	4.0 cm^2^ (4)

## Data Availability

The data presented in this study are available on request from the corresponding author. The data are not publicly available due to ethical considerations and data regulations.
